# Digital Image Forgery Detection Using JPEG Features and Local Noise Discrepancies

**DOI:** 10.1155/2014/230425

**Published:** 2014-03-16

**Authors:** Bo Liu, Chi-Man Pun, Xiao-Chen Yuan

**Affiliations:** Department of Computer and Information Science, University of Macau, Macau, China

## Abstract

Wide availability of image processing software makes counterfeiting become an easy and low-cost way to distort or conceal facts. Driven by great needs for valid forensic technique, many methods have been proposed to expose such forgeries. In this paper, we proposed an integrated algorithm which was able to detect two commonly used fraud practices: copy-move and splicing forgery in digital picture. To achieve this target, a special descriptor for each block was created combining the feature from JPEG block artificial grid with that from noise estimation. And forehand image quality assessment procedure reconciled these different features by setting proper weights. Experimental results showed that, compared to existing algorithms, our proposed method is effective on detecting both copy-move and splicing forgery regardless of JPEG compression ratio of the input image.

## 1. Introduction

Nowadays altering digital images via intuitive software is an operation of simpleness with very low cost; thus every individual can synthesize a fake picture. For the widely accessible Internet, the false information disseminates extremely fast. As a consequence, the facts may be distorted and the public opinion may be affected, yielding negative social influence. It can be even worse in the justice when pictures are presented as evidence. Therefore, there is a strong demand for valid and robust authentication method to discern whether a picture is original or not.

Two means are commonly utilized to make a forgery: copy-move and splicing. In the former case, a part of a picture is duplicated and then pasted onto other regions to cover any unwanted portion within the same picture [1]. In the latter case, tampered image consists of two sources and retains the majority of one image for detail [1]. Researchers and scientists have proposed many methods [2] to expose such intended manipulations. Passive forensic methods fulfill the task without additional information except for the image itself, thus showing advantages over active algorithms like watermarking and other signature schemas. Hence most research work is absorbed in developing blind authentication methods.

The forged picture leaves some clues which can be used to locate the manipulated regions. In the practice of copy-move operation, because the pasted area, though it may probably be altered geometrically, shares some similar features with the original region which is duplicated, searching for analogous features abstracted from local area is a possible solution. SIFT feature can be used to locate clone areas [3, 4]. For splicing tampered image detection, considering that there may be some discrepancies between the host image and the spliced region attempts to find the difference to expose that forgeries make sense. For instance, Kakar et al. [5] took advantage of motion blur discrepancy to detect fake pictures. For so many pictures stored or disseminated in JPEG format, some traces left by JPEG compression algorithm can be used. Estimating the quantization matrix used in JPEG compression, regions that possess inconsistent DCT coefficients are regarded as splicing area since an intact JPEG format picture should be coded by only one quantization table. Hamdy et al. developed this idea in [6]. However, this approach fails to deal with double compression. Indeed, this method is effective only on detecting BMP format image which is composed of two JPEG pictures with different compression table. Although complex situation in double compression was discussed [7], multicompressions more than twice are still very hard to analyze.

Our goal is to automatically detect copy-move as well as splicing forgery within a single process without any prior knowledge about the forgery type of the doubtful picture. The reason is obvious. Rather than putting the same picture into different algorithm, which may be effective on only one certain type of forgery, one single method is time saving and avoids evaluating every detection result, which will be very hard to discern the true output from various results. Lin and Wu in [8] proposed an integrated method to detect both copy-move and splicing forgery. But this method just connects two separate processes together. The errors in the step of forgery type judgment will greatly affect the detection result. Actually it is unnecessary to classify the pictures into certain forgery type if there is a tool or feature sensitive to two attack practices. Li et al. [9] proposed a method based on JPEG block artificial grids (BAG) detection to expose both splicing and copy-move forgeries. But there are two major problems in that paper; the first is that the algorithm must be adjusted before applying to different forgery; the author revised the splicing detection algorithm to deal with copy-move practice. However in practice we do not know the forgery type for a doubtful image. The other defect is that the algorithm is sensitive to highly compressed image and ineffective on high quality picture with little compression. To overcome this shortcoming, we inducted noise feature to compensate for BAG algorithm. When the picture is less compressed, clear BAG is very difficult to extract. We consider that local noise level and category can be used as a feature to identify different source of regions in a picture. Inconsistency and discrepancy from regions to regions provide the other clues except for BAG to locate forged area. Therefore, we created an integrated feature combining BAG and noise feature to verify the authenticity of a doubtful picture.

The paper is organized as follows.  Section 2 introduces the block artificial grids and noise patterns for detecting forgeries, and the following Section 3 details our proposed integrated method. In Section 4, experimental results will be presented to show the effectiveness of our method and we also compared it with existing methods. Finally we conclude this paper in Section 5.

## 2. Block Artificial Grid and Noise Estimation

### 2.1. Block Artificial Grid Extraction

**Figure 1:**
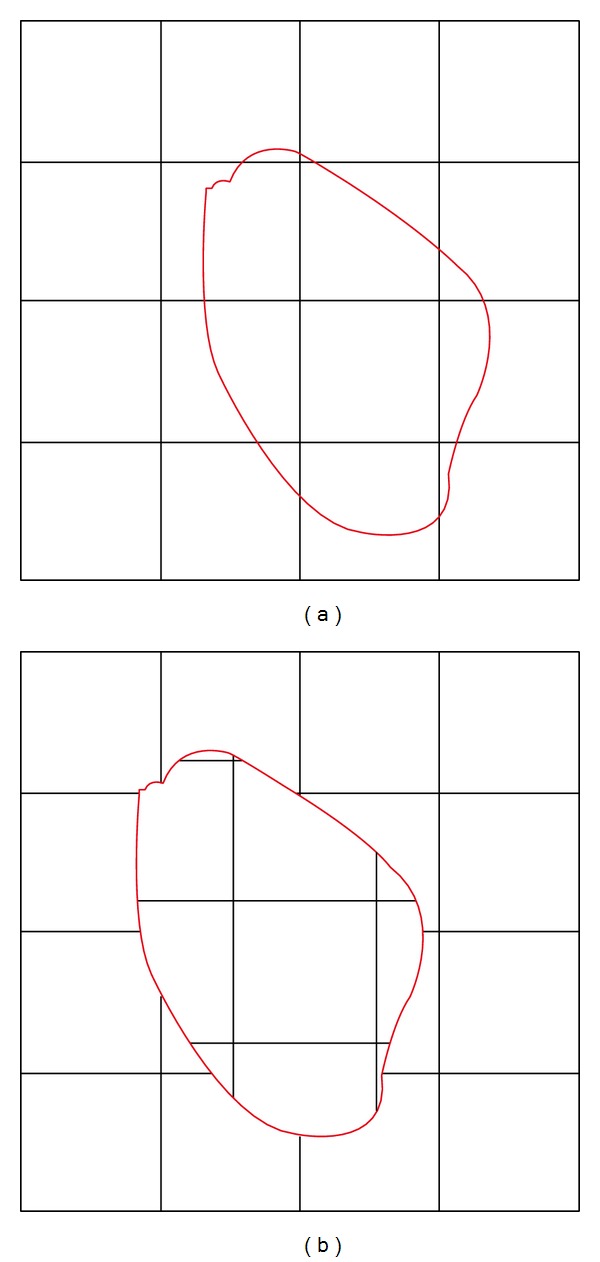
Illustration of BAG mismatch: the region within the red circle in upper left picture is copied and spliced into upper right picture. BAGs appearing within 8 × 8 block's border are suspected to belong to regions from other pictures. This mismatch may appear also in copy-move forgery practice.

It is universally known that the lossy JPEG compression will introduce some visually vertical or horizontal breaks in the image. These breaks called block artificial grid (BAG) appear at the border of each 8 × 8 pixel block. This property can be used to determine whether a picture is altered or not. If the picture is intact, block artificial grids should only present on block borders, while there is a great possibility that copied and pasted or spliced regions will bring their original BAGs which may appear within the 8 × 8 block rather than at borders. Some papers [9, 10] noticed this and Figure 1 illustrates the phenomenon. Theoretically speaking, if we extract all the BAGs from a given image, areas with BAGs within the block border are regarded as forged regions. Li et al. [9] introduced steps to extract BAGs. As it is mentioned before, artificial grids are visually vertical and horizontal lines, and they are very weak when comparing to the border lines of objects in the picture. And the main purpose of extraction procedures is to enhance these weak lines and to make them visible. However lines are also strengthened which may be the edges of objects or just objects themselves. This will interfere with the detection result because we only need BAGs. To allay the side effect, we preprocessed the doubtful image by excluding the edges of objects. But it should be noticed that BAG can also be regarded as vertical or horizontal edges. For preserving BAGs we only excluded edges within certain range.

Suppose that *G* was the grayscale version of image *I* and then the edge *E* was obtained by *E* = *G*∗*S*, where *S* represents Sobel operator and “∗” denotes convolution. Then we defined whether a pixel is excluded using

(1) $$\begin{array}{c} R\left(m,n\right)=\{\begin{array}{cc} 0 & D\left(E\left(m,n\right)\right)\in \left[0,\theta \right]\\ & \cup \left[\frac{\pi }{2}-\theta ,\frac{\pi }{2}+\theta \right]\cup \left[\pi -\theta ,\pi \right),\\ 1 & \text{others}, \end{array} \end{array}$$

where *D*(·) denotes gradient of the pixel and *R*(*m*, *n*) = 1 means excluded pixels. Then we begin to extract BAGs.

Firstly weak horizontal edges were extracted by calculating second-order difference of an image. For the test image, *I*(*m*, *n*), absolute second-order difference *d*(*m*, *n*) was obtained by

(2) $$\begin{array}{c} d\left(m,n\right)=\left|2I\left(m,n\right)-I\left(m+1,n\right)-I\left(m-1,n\right)\right|. \end{array}$$

Then all differentials larger than 0.1 or *R* = 1 are discarded. In subsequence, enlarged horizontal lines are accumulated from every 33 columns, as shown in (3). Then a median filter Med[·] is used to refine the result in

(3) $$\begin{array}{c} a\left(m,n\right)=\overset{16}{\underset{i=n-16}{\sum }}d\left(m,i\right). \end{array}$$

(4) $$\begin{array}{c} a_{r}\left(m,n\right)=a\left(m,n\right)-\text{Med}\left[\left\{a\left(i,n\right)\;|\;m-16\leq i\leq m+16\right\}\right]. \end{array}$$

Weak horizontal edge *b*_*h*_ is further periodical median filtered as

(5) bh(m,n)=Med[{ar(i,n) ∣ i=m−16,m−8,m,m+8,m+16}].

Similarly, the vertical BAGs *b*_*v*_ can also be attracted. As a result, final BAG is obtained by adding two components together in

(6) $$\begin{array}{c} b\left(m,n\right)=b_{h}\left(m,n\right)+b_{v}\left(m,n\right). \end{array}$$

### 2.2. Noise Estimation

**Figure 2:**
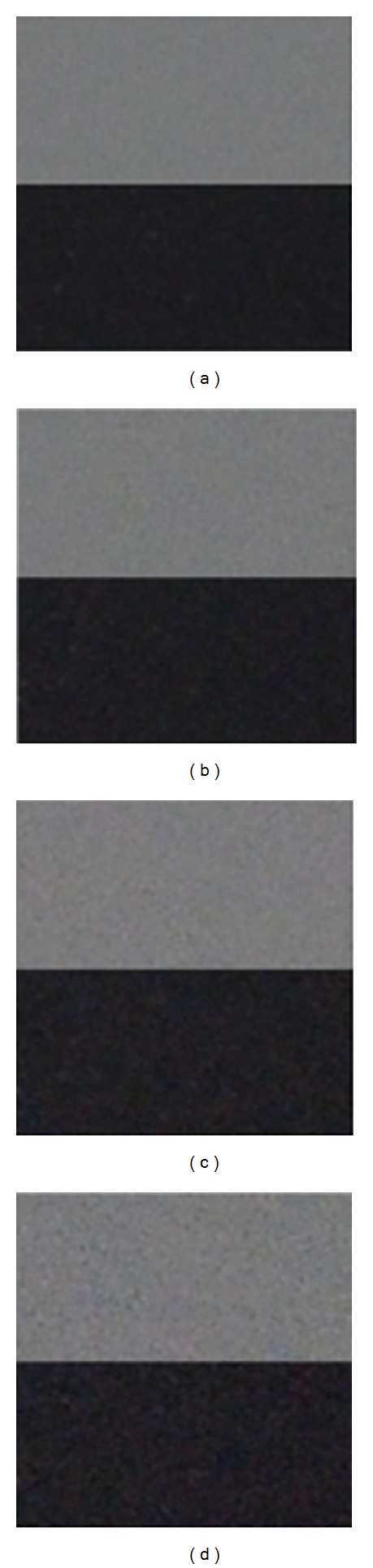
Visual noise comparison for pictures captured by the same camera Nikon D7000 under the same scenery with different ISO settings: (a) ISO = 100, (b) ISO = 800, (c) ISO = 1600, and (d) ISO = 3200. Crops are 100% with ambient temperature approximately 22C.

**Figure 3:**
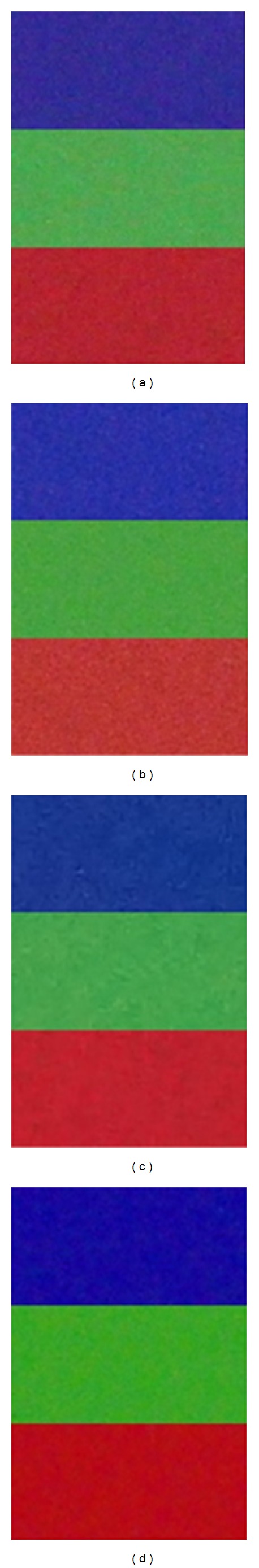
Visual noise comparison for pictures taken by different cameras under the same scenery with ISO = 1600: (a) Canon 550D, (b) Nikon D7000, (c) Sony A77, and (d) Pentax K5.

Highly compressed by JPEG, the picture shows visual block artificial grids across the whole frame which can be extracted by algorithm described in Section 2.1. However, under some circumstances, when the picture is not highly compressed and stored in high quality, the way by using BAG only becomes harder to detect forgery. To increase the versatility of the algorithm, we use noise feature. The noise comes from imaging sensor and internal circuits within a camera. And the number of noise changes in accordance with camera settings especially ISO sensitivity and exposure time. As an example, Figure 2 shows that the visual noise of images is captured from a Nikon D7000 camera. We can see that more noise appears in the image as the ISO speed rises. In Figure 3 we can see that different camera model from different manufacture also shows unequal noise amount and forms although the pictures were taken in the same scenery with equal ISO speed. So the noise can be used to help distinguish difference sources of a picture. When two pictures are spliced together the noise level or patterns are inconsistent between regions. By estimating the pattern or level of noise in different regions the forgery can be exposed via noise discrepancies.

In most cases, the alien region has a specific shape, such as a tree, a bird, or a person. The forged object may possess different noise level compared to that of its surroundings. To estimate every region's noise level, the image should be firstly divided into small segments. Most previous methods divide the picture into small overlapping blocks with equal size. But, in our application, this will lead to bad performance in next steps which need accurate noise estimation of each region to compare noise discrepancy. This is because the forged area is not rectangle in most cases, and the small block will contain original and alien pixels. Therefore we segment picture into sets of pixels, not regular shaped, also known as superpixels. Employing this approach makes segments more meaningful and easier to be processed in the following steps because the segmentation algorithms locate the objects and boundaries other than the same-size blocks. The result of image segmentation is a set of segments that collectively cover the entire image or a set of contours extracted from the image. Each of the pixels in a region is similar with respect to some characteristic or computed property, such as color, intensity, or texture. Adjacent regions are significantly different with respect to the same characteristic(s) [11].

In our application, SLIC (simple linear iterative clustering) superpixels algorithm [12] was used to segment picture. This algorithm is easy but better than other segmentation methods. Given an *M* × *N* image *I*_*s*_^*c*^ where *c* ∈ {red, green, blue} denotes different color channel. The meaning of subscript *s* will be explained later and

(7) $$\begin{array}{c} I=\left(\begin{array}{ccc} I_{s}^{c}\left(1,1\right) & \cdots & I_{s}^{c}\left(1,N\right)\\ \vdots & \ddots & \vdots \\ I_{s}^{c}\left(M,1\right) & \cdots & I_{s}^{c}\left(M,N\right) \end{array}\right). \end{array}$$

In essence, SLIC is a clustering algorithm. Similar to other clustering methods, two steps are evolved with SLIC segmentation. In the initialization step, cluster centers *C*_*s*_ = (*l*_*s*_,*a*_*s*_,*b*_*s*_,*x*_*s*_,*y*_*s*_)^*T*^ are assigned by sampling pixels at regular grid. Note that the picture is segmented in LAB color space. Then cluster centers are moved to the lowest gradient position in a 3 × 3 neighborhood. In the assignment step, each pixel is associated with the nearest cluster center and an update step adjusts the cluster centers to be the mean (*l*,*a*,*b*,*x*,*y*)^*T*^ vector of all the pixels belonging to the cluster. A residual error *E* between the new and previous cluster center locations is computed. Once *E* ≤ *threshold* the algorithm stops. We assigned subscript *s* which denoted segment number to every pixel.

Before construction of noise feature for every segment, we excluded sharp transitional area since noise estimation was adversely affected by heterogeneous image content [13]. We estimated sharp area using its gray-scale image *G* which was calculated by

(8) $$\begin{array}{c} G=0.2989I^{r}+0.5870I^{g}+0.1140I^{b}. \end{array}$$

The sharpness edge of image was then obtained by *E* = *G*∗*S*, where *S* represents Sobel operator and “∗” denotes convolution. We then define whether a pixel is in the sharp area using

(9) $$\begin{array}{c} H\left(m,n\right)=\{\begin{array}{cc} 1, & \left(m,n\right)\in E,\\ 0, & \left(m,n\right)\notin E, \end{array} \end{array}$$

where *H*(*m*, *n*) = 1 means that the pixel (*m*, *n*) is located in sharp transitional area. To guarantee that these areas will not affect noise estimation in the next step, we expand boundaries via dilation by

(10) $$\begin{array}{c} Z=H⊕V, \end{array}$$

where *V* is a structure element of 3 × 3 ones. *Z* is the expanded sharp area.

To extract noise feature of each segment produced by previous SLIC algorithm, we firstly employed denoising algorithm across the whole picture. The estimated noise *f* at location (*m*, *n*) of image *I* ^*c*^ was calculated by

(11) $$\begin{array}{c} f^{cd}\left(m,n\right)=I^{c}\left(m,n\right)-H^{cd}\left(m,n\right), \end{array}$$

where *H* ^*cd*^ = *I* ^*c*^∗*P* ^*d*^ and filter *P* ^*d*^, *d* = 1,2,…, 5 represents five different filters used to trace different aspects of the noise [14]. They are median filter, Gaussian filter, averaging filter, and adaptive Wiener denoising with two neighborhood sizes 3 × 3 and 5 × 5, respectively. For instance, high frequency noise can be detected by using Gaussian filter and median filter addresses “salt and pepper” noise.

For each combination of color channel *c* and denoising filter *P* ^*d*^ we calculated the mean and standard deviation *σ*_*s*_^*cd*^ values of each segment *s* as the noise feature *F*_*s*_^*cd*^ = (*μ*_*s*_^*cd*^, *σ*_*s*_^*cd*^), where

(12) $$\begin{array}{c} \mu_{s}^{cd}=\frac{1}{R}\sum_{Z(m,n)=0}f_{s}^{cd}\left(m,n\right),\\ \sigma_{s}^{cd}=\frac{1}{R}{\left(\sum_{Z(m,n)=0}{\left(f_{s}^{cd}\left(m,n\right)-\mu_{s}^{cd}\right)}^{2}\right)}^{1/2}. \end{array}$$

As a result we computed 3 × 5 × 2 = 30 dimensional feature vector *F*_*s*_ of a segment.

## 3. Integrated Method for Forgery Detection

**Figure 4:**
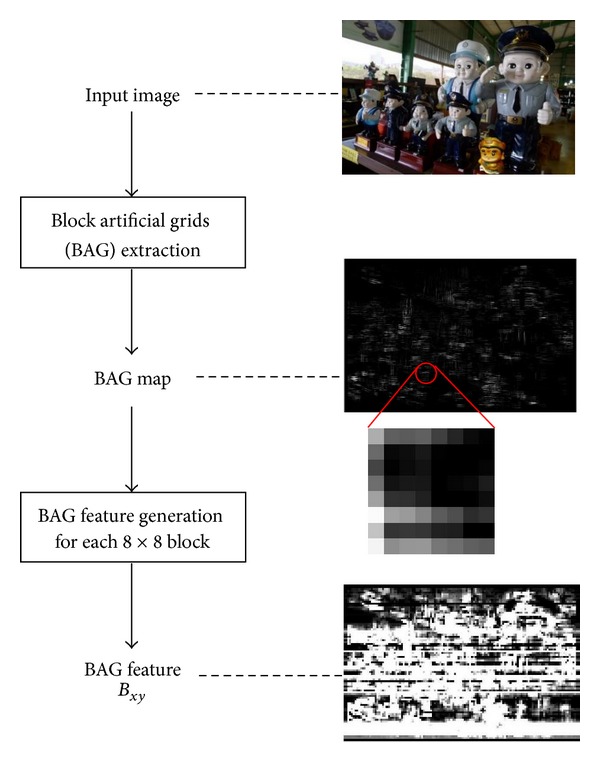
Flowchart of proposed method: BAG feature generation.

**Figure 5:**
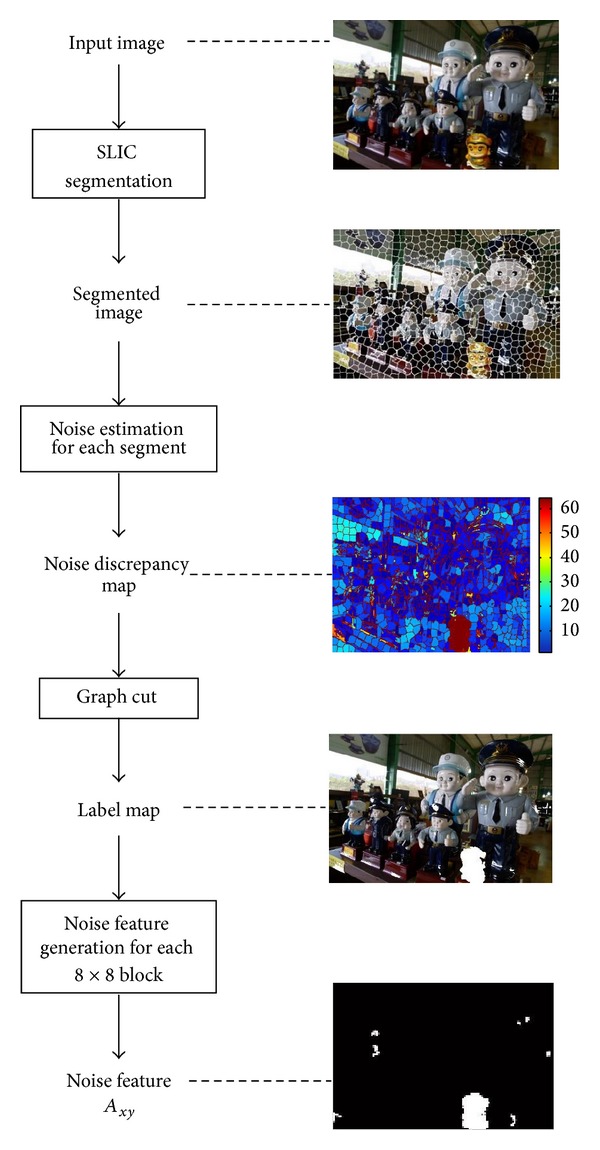
Flowchart of proposed method: noise feature generation.

**Figure 6:**
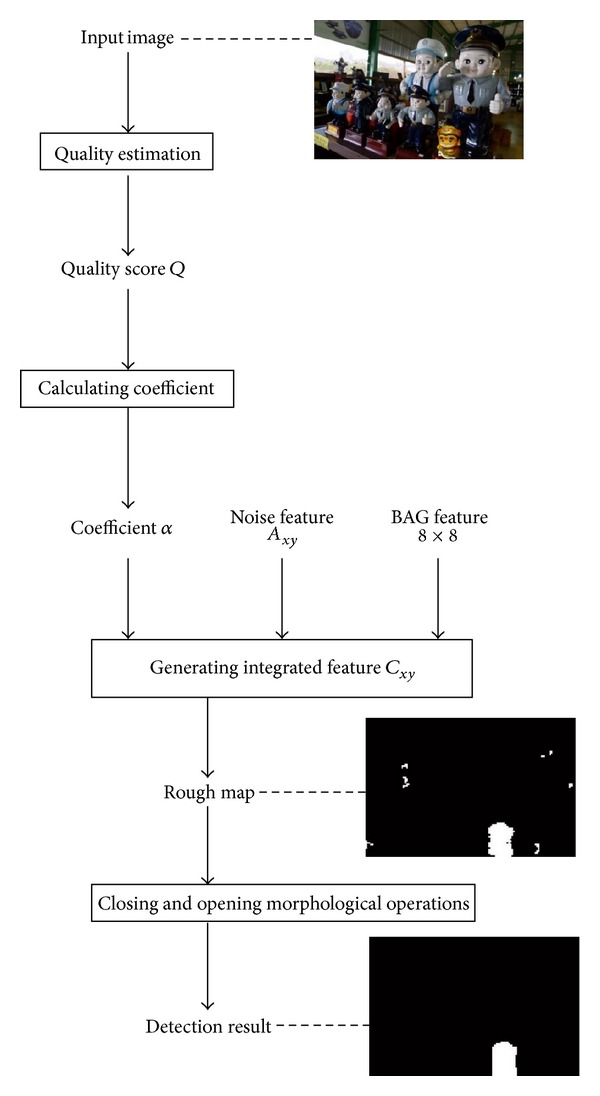
Flowchart of proposed method: combination of two features.

We proposed an integrated method effective to both copy-move and splicing forgery. Based on combination of block artificial grid extraction with analysis of local noise discrepancies, the algorithm showed valid performance to high compression JPEG pictures, as well as high quality images lack of BAGs. To implement the authentication process, we built an indicator for every 8 × 8 nonoverlapped blocks of the doubtfulful picture. The indicator mathematically described the possibility of the block being a forged area, with higher value denoting higher probability. As described in Figures 4, 5, and 6, for each 8 × 8 block, BAG feature and assigned label based on noise discrepancies were integrated by estimated compression indicator. (see Algorithm 1 for a detailed description).

### 3.1. Block BAG Feature Construction

In Section 2.1 we introduced how to extract BAGs. For an intact picture, the BAGs appear at the border of each 8 × 8 block, while for a picture with intentional copy-move or splicing operation some BAGs will be presented at some abnormal positions, such as the center of the block. For a fixed 8 × 8 block *I*_*xy*_, these abnormal BAGs can be calculated [9] by

(13) Bxy=Max⁡{∑i=27b(i,n) ∣ 2≤n≤7}−Min⁡{∑i=27b(i,n) ∣ n=1,8}+Max⁡{∑i=27b(m,i) ∣ 2≤m≤7}−Min⁡{∑i=27b(m,i) ∣ m=1,8}.

### 3.2. Noise Discrepancy Label Assignment

The noise feature of each segment had been calculated in Section 2.2 and then our goal was to segment the image into two regions with noise discrepancies. To achieve the target the energy-based graph cuts can be used.

**Figure 7:**
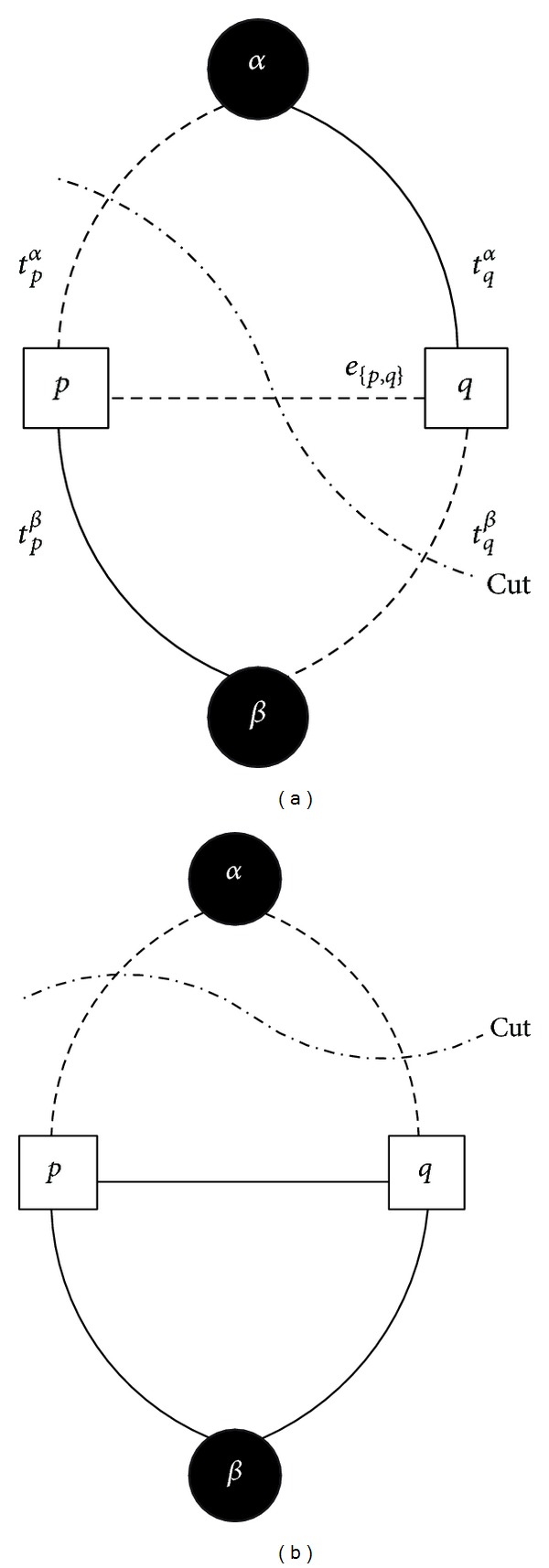
Two possible graph cuts result. *αβ* are two labels and *pq* are pixels.

Energy minimization via graph cuts is proposed by Boykov et al. [15] to solve labeling problems with low computation cost. In a common label assignment problem, the labels should vary smoothly almost everywhere while preserving sharp discontinuities existing at object boundaries. These two constraints can be formulated as *E*(*f*) = *E*_smooth_(*f*) + *E*_data_(*f*), where *f* is a labeling that assigns each pixel *p* ∈ *P* a label *f*_*p*_ ∈ *ℒ* and *E*_smooth_ measures the extent to which *f* is not piecewise smooth while *E*_data_ measures the disagreement between *f* and observed data. The goal is to minimize the function. Specifically the energy function can be rewritten as

(14) $$\begin{array}{c} E\left(f\right)=\underset{\left\{p,q\right\}\in N}{\sum }V_{p,q}\left(f_{p},f_{q}\right)+\underset{p\in P}{\sum }D_{p}\left(f_{p}\right), \end{array}$$

where *N* is neighboring pixels, *V* is the penalty of pairs in the first term, and *D*_*p*_ is nonnegative and measures how well label fits pixel. Local minimum value can be obtained with the help of graph cuts. The simplified problem is illustrated in Figure 7. Since many algorithms have been proposed to solve min-cut problem, if proper weight value is assigned to each edge, the problem of minimizing energy function changes to min-cut problem. The weight is seen in Table 1. The calculation result is a cut *C* which separates two labels. Figure 7 shows two possible cuts and the label is assigned to the pixel when cut *C* contains the edge connecting that label to the pixel. For example, in left case of Figure 7, label *α* is assigned to pixel *p* while *β* is assigned to *q* because cut *C* contains edge *t*_*p*_^*α*^ and *t*_*q*_^*β*^.

Our forgery detection task can also be regarded as a labeling problem. In our application, there are two labels that need to be assigned to each segment produced by previously introduced SLIC algorithm: forged area as they show inconsistency to rest segments in terms of noise level or pattern and the original area. And each segment is processed as a pixel. The reason why we avoid employing widely used outlier detection algorithms [16] and Otsu's automatic thresholding method [17] is the property of noise. From Figure 2 we observe that even the picture is taken by one camera and the amount of noise differs in different illumination. The color of object may also affect the noise level. Accordingly the ideal algorithm should tolerate these local deviations and inconsistencies. In other words, it should keep “smooth” across the image while preserving “sharp” discontinuity in inconsistent boundaries. This requirement is identical to label assignment problems described previously while normal outlier detection algorithms are not capable of this.

“Smooth” constraint is realized by proper assignment of *V*(*α*, *β*) and “sharp” discontinuity requirement is supported by *D*_*p*_(∗). We firstly discuss the weight of edge *t*_*p*_^*α*^ and *t*_*p*_^*β*^. We computed average value of feature vector of all segments in 30 dimensions and named it the mean vector $\overset{-}{F}$. Then we found the vector whose Euclidean distance was the largest from $\overset{-}{F}$ by searching for all segments and called it *F*_max⁡_. For a feature vector *F*_*s*_ the weight *w* was obtained by

(15) $$\begin{array}{c} w_{\alpha }=||F_{s}-\overset{-}{F}||;\;\;w_{\beta }=||F_{s}-F_{\max}||, \end{array}$$

where *α* was “original” label while *β* was “forged” and ||·|| denoted Euclidean distance between two vectors.

From (15) we can find that, if the noise level of a segment is close to the average value across the whole picture, the weight *w*_*α*_ assigned is small while *w*_*β*_ is large and vice versa. This meets the requirement of discontinuity preserving. Then it is the turn to discuss smooth constraint. Proper value of interaction penalty *V*(*α*, *β*) tolerates local deviations of noise which is affected by illumination or color. There are many forms proposed. For an instance, *V*(*α*, *β*) = min⁡(*K*, |*α* − *β*|) or an important function given by the Potts model *V*(*α*, *β*) = *K* · *T*(*α* ≠ *β*) where *T*(·) is 1 if its argument is true and otherwise 0. This penalty function possesses good feature of piecewise smooth, so we used it in the experiment.

Graph cut based on noise discrepancy assigned every segment *S*_*k*_ a label *ℒ* ∈ {0,1} indicating whether the area was classified as forged (*ℒ*_*k*_ = 1) or not (*ℒ*_*k*_ = 0). And we assigned every pixel belonging to the segment the same label *ℒ*(*m*, *n*) = *ℒ*_*k*_, (*m*, *n*) ∈ *S*_*k*_. At last, block indicator *A*_*xy*_ described the possibility of forgery and was calculated by

(16) $$\begin{array}{c} A_{xy}=\frac{1}{64}\overset{8y}{\underset{j=8y-7\;\;}{\sum }}\overset{8x}{\underset{i=8x-7}{\sum }}L\left(i,j\right). \end{array}$$

### 3.3. Feature Generation for Forgery Detection

In this step, we combined together two features described already with proper coefficient. Since the method based on BAG extraction is only sensitive and feasible to highly compressed images, the form of combined feature is described as *C*_*xy*_ = *αB*_*xy*_ + (1 − *α*)*A*_*xy*_ and *α* denotes the coefficient assigned and is a function of evaluated image compression rate or the JPEG image quality; namely, *α* = *f*(*Q*).

**Figure 8:**
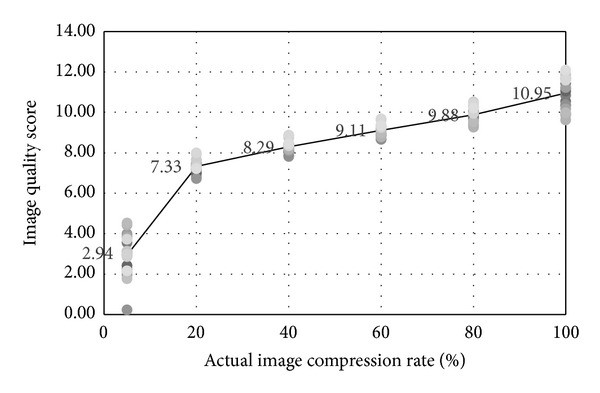
Image quality score in different compression rate: the numbers in the figure denote average value of quality scores of 20 pictures in the same compression rate.

We firstly evaluated the quality of the picture and then found the function *f*. Proposed by Wang et al. [18], the quality assessment algorithm is nonreferenced and sensitive to JPEG compression rather than noise which was tested and verified by our experiment. We took 20 pictures in raw file (no compression) and then saved them as JPEG format pictures with different compression ratio. In our experiment, 100% means saving with the highest quality and the lowest compression. We assessed the picture quality of compression rate as 100%, 80%, 60%, 40%, 20%, and 5%, respectively, and averaged the scores. See Figure 8 for result: less compressed pictures show higher quality scores.

**Figure 9:**
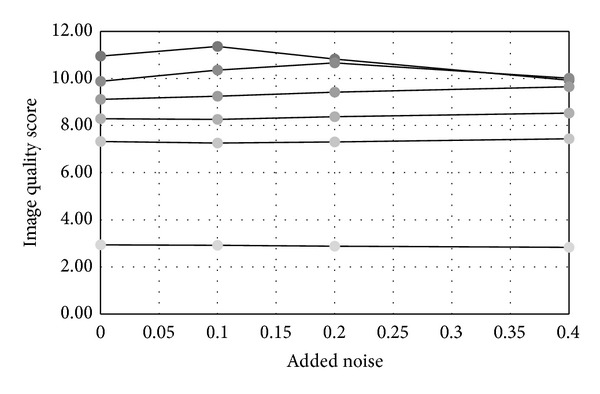
Image quality score in different noise level.

However, the algorithm is less sensitive to noise effect. In the experiment, for each image set with certain compression rate, we added 10%, 20%, and 40% monologue Gaussian noise to the image, respectively, and then obtained the average quality scores. See Figure 9 for result: noise does not largely affect quality scores. Therefore, we consider that the dominated factor affecting quality score in algorithm [18] is JPEG compression rate.

Then we discuss how to generate the function *α* = *f*(*Q*). In the experiment, we made 60 fake pictures and every 10 pictures were compressed in a certain rate. And then we used BAG feature only to detect the forgeries.  Table 2 shows detection accuracy in different compression rate. The experimental result confirmed that the BAG method is good at dealing with the pictures with low quality score. Therefore, the value of *α* should approach 1 when *Q* declines near to 2 for its detection accuracy is 100%. Meanwhile, *α* should be set to 0 when *Q* rises to 9 or so because of its low accuracy.

The function *f* we recommended based on experimental result is

(17) f(Q) ={1,Q<2,−0.0213Q+1.0469,2≤Q<6.9,−0.2980Q2+4.2584Q−14.2952,6.9≤Q<8.9,0,Q≥8.9.

In order to filter out some isolated false marked areas and improve the integrity of suspect forged region, morphological operations including closing and opening are used. The final result comes from (*C*•*M*_*c*_)∘*M*_*o*_, where *M*_*c*_ and *M*_*o*_ are circular structure with radius of 5 and 3 pixels, respectively.

## 4. Experimental Results and Discussion

This part firstly exhibits the experimental results and compares our results with existing algorithm. Then we consider the situation when the input image is slightly compressed. In this circumstance, there are few conspicuous block artificial grids; noise feature becomes predominated since *α* = *f*(*Q*) = 0. In order to verify the effectiveness of the proposed method, we tested under two situations noise discrepancies from artificial added noise and from digital cameras.

### 4.1. Detection Results and Comparison

**Figure 10:**
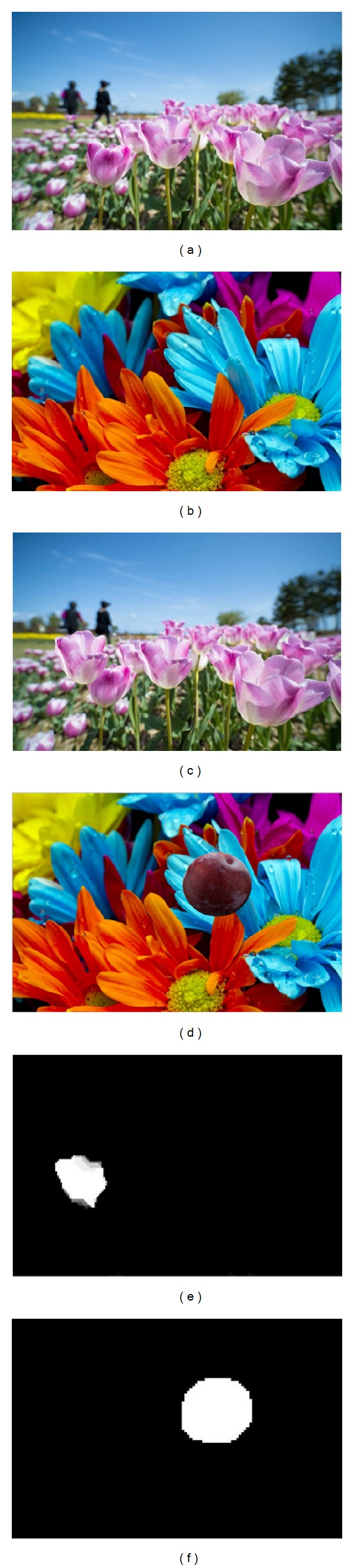
The detection result of copy-move and splicing forgeries: (a) and (b): intact pictures; (c) and (d): forged pictures; and (e) and (f): detection result.

As it is mentioned at the beginning, our proposed method can deal with both copy-move and splicing forgery with one authentication process. Two detection results are shown in Figure 10: the marked white area is detected forged region. Our algorithm shows good performance in these two types of forgery.

**Figure 11:**
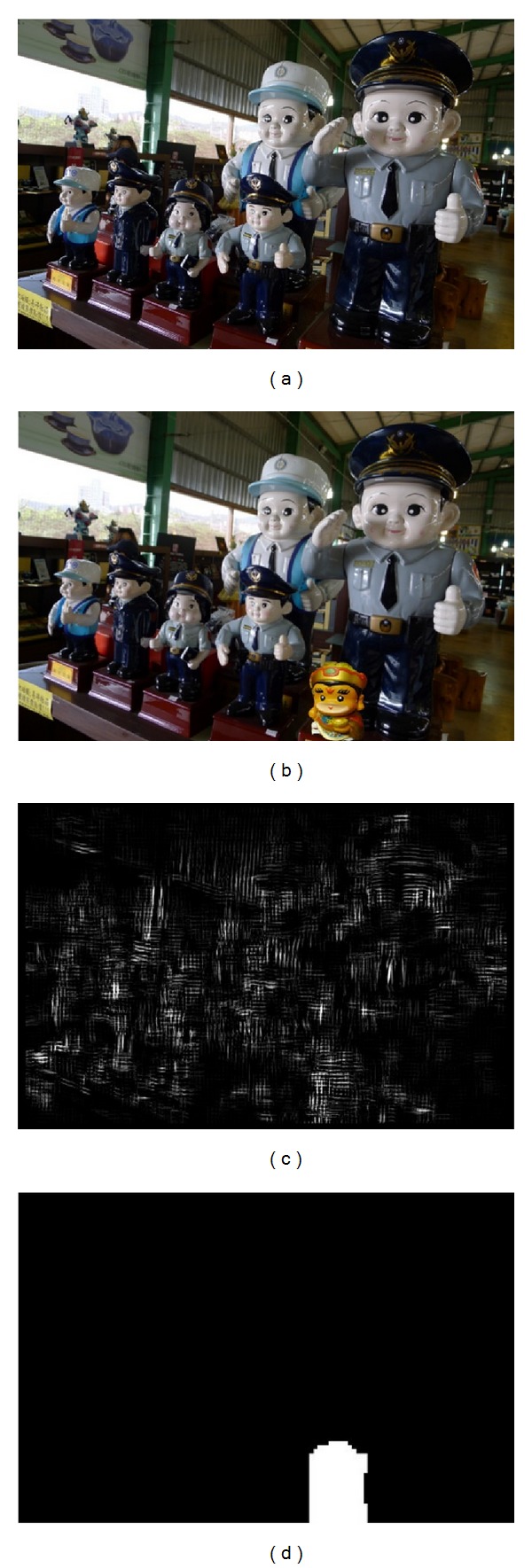
Comparison between the proposed method and Li's algorithm: (a)intact image; (b) picture with splicing forgery; (c) detection result of Li's algorithm; and (d) result of our method.

Then we compared our proposed method with existing algorithm in [9]. In the experiment, we prepared six sets of test images. In each set, there were 25 pictures including intact and fake pictures with copy-move or splicing forgery. The difference between sets was the image quality-JPEG compression rate.  Table 3 shows the comparison of detection accuracy between two methods; when the image is greatly degraded by high JPEG compression, two methods present valid performance. However, if the forged image is saved with slightly compression, the detection accuracy of Li's method drops significantly, while our method still maintains high accuracy.  Figure 11 shows an instance of detection result comparison between two methods.

### 4.2. Simulation Results

**Figure 12:**
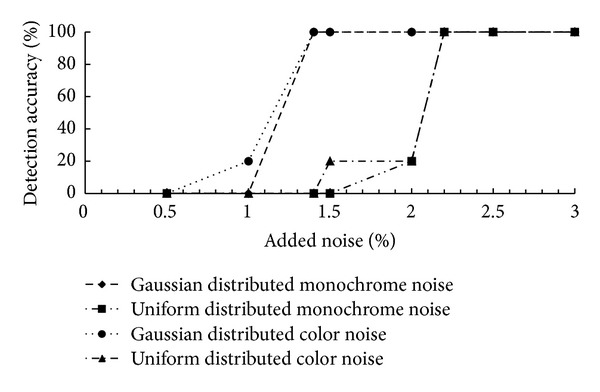
Finding lower limit amount of added noise that the algorithm can detect.

In this part we present a simulated forgery case that the noise is added to implanted region. This simulation also reflects a real splicing attack that in order to make the alien area visually resemble the rest part of picture noise may be applied. Since Photoshop is a popular image editing tool, we add noise to picture with provided filters by software. There are two noise distribution options: Gaussian and uniform and two noise patterns: monochrome and colored. Therefore four combinations are available and the user can alter the noise amount in percentage. The experiment is designed to demonstrate the sensibility of algorithm which is the lower limit amount of added noise that can be detected by our method.  Figure 12 shows the detection accuracy of four groups, each of which contains five forged pictures. We conclude that the effective lower limit for detection is 1.4% for Gaussian noise and 2.2% for uniform noise regardless of monochrome or colored noise pattern.

### 4.3. ISO and Detection Results

Two image datasets are prepared to verify the effectiveness of our proposed method. In the first set, all source pictures were taken by a Nikon D7000 DSLR camera and used to make splicing forgeries in combination of different ISO speed seen in Table 4. There are 10 forged pictures in the test set. The data in this table is the detection accuracy or true positive rate.

**Figure 13:**
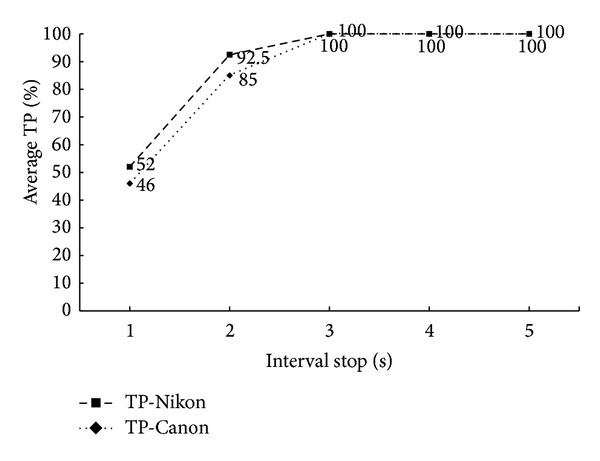
TP rate in different interval stop(s).

The ISO speed setting in camera is discrete without the same interval and we find that the higher TP rate appears at combination of two ISO speeds with big gap. In order to see this phenomenon clearly, we can see Figure 13. The horizontal axis is marked by interval stop(s) which denotes the interval ISO speed. For instance, the interval stop of ISO 100 and 200 is 1; this is the same with ISO 1600 and 3200, while that of ISO 200 and 1600 is 3. The average TR rate is calculated from Tables 4 and 5. We conclude that our method shows good performance in two or more interval stops.

**Figure 14:**
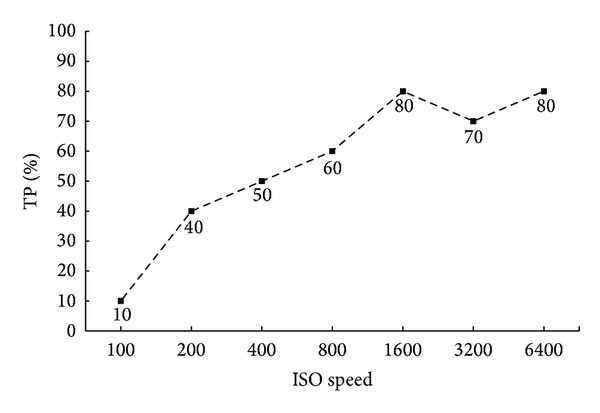
TP rate in different ISO speed.

The second experiment is to verify the effectiveness of detecting forgery in pictures combined from two different cameras. And in the paper we just show an extremely hard situation when the source pictures are taken in the same ISO speed. Two cameras are Nikon D7000 and Canon 550D, respectively. And 10 forged images in the set are used to the test. The TP rate is shown in Figure 14. And the accuracy increases as the ISO speed rises. The reason is that the image processing ability of two camera models is not the same. In lower ISO speed, less noise appears in the picture and this processing difference is small; therefore the TP rate is very low at 10%, while in high ISO settings, the method shows effectiveness again. Note that, in real situation, the ISO of two source pictures may not be the same; only one interval stop will highly enhance the accuracy as it is shown in the first experiment.

## 5. Conclusions

In this paper, we concentrated on exposing the two main types of image manipulation, copy-move and splicing forgery. We proposed an integrated algorithm to locate forged regions by a single authentication process. In our method, JPEG block artificial grids and local noise discrepancies were used to generate features which were combined with image quality score as coefficient. Experimental result shows that our approach is valid to both highly compressed and high quality pictures. Comparing to existing algorithms, our method has competitive advantages and a larger range of application.

## Figures and Tables

**Algorithm 1:**
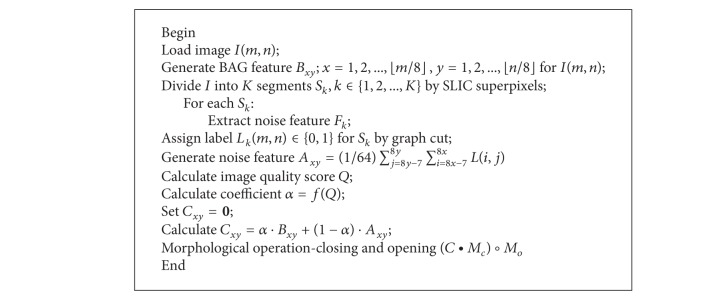
Algorithm description.

**Table 1:** Edge weights for graph cuts.

Edge	Weight	For
*t*_*p*_^*α*^	*D*_*p*_(*α*) + $\sum_{q\notin P_{\alpha \beta }}^{q\in N_{p}}V(\alpha ,\;f_{q})$	*p* ∈ *P*_*αβ*_
*t*_*p*_^*β*^	*D*_*p*_(*β*) + $\sum_{q\notin P_{\alpha \beta }}^{q\in N_{p}}V(\beta ,\;f_{q})$	*p* ∈ *P*_*αβ*_
*e*_{*p*,*q*}_	*V*(*α*, *β*)	$\begin{array}{c} \{p,q\}\in N\\ p,q\in P_{\alpha \beta } \end{array}$

**Table 2:** Detection accuracy in different image quality.

Compression rate	Quality score *Q*	Accuracy
5%	2.24	100%
20%	6.84	90%
40%	7.90	70%
60%	8.88	60%
80%	9.68	0%
100%	10.57	0%

**Table 3:** Comparison between two methods.

Set	Compression ratio	Accuracy of Li [9]	Accuracy of our method
1	100%	0%	80%
2	80%	0%	84%
3	60%	56%	80%
4	40%	84%	88%
5	20%	92%	92%
6	5%	100%	100%

**Table 4:** Combination of ISO speed and respective TP rate. Source pictures are taken by Nikon D7000.

ISO	100	200	400	800	1600	3200
100	—*	20	90	100	100	100
200	20	—	40	80	100	100
400	90	40	—	50	100	100
800	100	80	50	—	80	100
1600	100	100	100	80	—	70
3200	100	100	100	100	70	—

**Table 5:** Combination of ISO speed and respective TP rate. Source pictures are taken by Canon 550D.

ISO	100	200	400	800	1600	3200
100	—*	30	80	100	100	100
200	30	—	20	80	100	100
400	80	20	—	40	90	100
800	100	80	40	—	60	90
1600	100	100	90	60	—	80
3200	100	100	100	90	80	—

*not verified in experiment.
